# Dyrk1A overexpression leads to increase of 3R-tau expression and cognitive deficits in Ts65Dn Down syndrome mice

**DOI:** 10.1038/s41598-017-00682-y

**Published:** 2017-04-04

**Authors:** Xiaomin Yin, Nana Jin, Jianhua Shi, Yanchong Zhang, Yue Wu, Cheng-Xin Gong, Khalid Iqbal, Fei Liu

**Affiliations:** 1 Department of Neurochemistry, Inge Grundke-Iqbal Research Floor, New York State Institute for Basic Research in Developmental Disabilities, Staten Island, New York 10314 USA; 2 Key Laboratory of Neuroregeneration of Jiangsu and Ministry of Education, Co-innovation Center of Neuroregeneration, Nantong University, Nantong, Jiangsu 226001 P. R. China; 3 Department of Pharmacology, Xuanwu Hospital of Capital Medical University, Beijing, 100053 P. R. China

## Abstract

Alternative splicing of tau exon 10 generates tau isoforms with three or four microtubule-binding repeats, 3R-tau and 4R-tau, which is equally expressed in adult human brain. Imbalanced expression in 3R-tau and 4R-tau has been found in several sporadic and inherited tauopathies, suggesting that dysregulation of tau exon 10 is sufficient to cause neurodegenerative diseases. We previously reported that Dyrk1A, which is overexpressed in Down syndrome brains, regulates alternative splicing of exogenous tau exon 10. In the present study, we investigated the regulation of endogenous tau exon 10 splicing by Dyrk1A. We found that inhibition of Dyrk1A enhanced tau exon 10 inclusion, leading to an increase in 4R-tau/3R-tau ratio in differentiated-human neuronal progenitors and in the neonatal rat brains. Accompanied with overexpression of Dyrk1A, 3R-tau was increased and 4R-tau was decreased in the neonatal brains of Ts65Dn mice, a model of Down syndrome. Treatment with Dyrk1A inhibitor, green tea flavonol epigallocatechin-gallate (EGCG), from gestation to adulthood suppressed 3R-tau expression and rescued anxiety and memory deficits in Ts65Dn mouse brains. Thus, Dyrk1A might be an ideal therapeutic target for Alzheimer’s disease, especially for Down syndrome and EGCG which inhibits Dyrk1A may have potential effect on the treatment or prevention of this disease.

## Introduction

Tau is the major neuronal microtubule associated protein and plays a crucial role in the maintenance of both the neuronal cytoskeleton and axonal transport. Tau is hyperphosphorylated and aggregated into neurofibrillary tangles (NFT) in the affected neurons in the brain of patients with Alzheimer disease (AD)^1, 2^ and other related neurodegenerative disorders with tau pathology called tauopathies. The correlation between the numbers of NFTs in brain and the severity of dementia symptoms in the patients^3–5^ indicates a pivotal role of tau pathology in AD.

Adult human brain expresses six isoforms of tau from a single gene by alternative splicing of the exons 2, 3 and 10 of its pre-mRNA^6–8^. Exon 10 encodes the second microtubule-binding repeat. Its alternative splicing generates tau isoforms containing either three or four microtubule-binding repeats, named 3R-tau and 4R-tau, respectively^7^. Alternative splicing of exon 10 is developmentally regulated — fetal or neonatal human brain only expresses 3R-tau, whereas adult human brain expresses equal levels of 3R-tau and 4R-tau^7, 9^. Many mutations in tau gene associated with frontotemporal dementia with Parkinsonism linked to chromosome 17 (FTDP-17) cause dysregulation of tau exon 10 splicing but not protein sequence, leading to a selective increase in either 3R- or 4R-tau^10^. Moreover, imbalanced expression in 3R-tau and 4R-tau has also been seen in other tauopathies, including Pick’s disease, progressive supranuclear palsy, and cortico-basal degeneration. Thus, equal levels of 3R-tau and 4R-tau are critical for maintaining optimal neuronal physiology in human brain^10^. Dysregulation of tau exon 10 itself is sufficient to cause neurofibrillary degeneration.

Down syndrome (DS) is a genetic disorder caused by the presence of all or part of the third copy of chromosome 21. By the age of 40’s, the brains of almost all individuals with DS develop typical AD pathologies, amyloid plaques and NFTs^11, 12^. Aβ pathology may be caused by overexpression of APP as a result of the extra copy of the gene. However, the molecular mechanism leading to early tau pathology in DS remains unclear.

*Dyrk1A* (dual-specificity tyrosine-phosphorylated and regulated kinase 1A) encodes a proline/arginine-directed serine/threonine kinase^13^ and is located on the Down syndrome critical region. Dyrk1A is overexpressed in individuals with DS due to an extra copy of the gene. As a protein kinase, Dyrk1A phosphorylates numerous proteins and involves in the regulation of multiple cellular function. Dyrk1A contains two nuclear localization signaling and is mainly expressed in nucleus and co-localized with splicing factor SC35 in nuclear speckle compartment^14^. Dyrk1A phosphorylates SF3b1/SAP155^15^ and cyclin L2^16^, suggesting Dyrk1A may regulate the splicing by phosphorylation of the splicing factors. We recently found that Dyrk1A phosphorylates splicing factors, alternative splicing factor/splicing factor 2 (SF2/ASF), 9G8, SC35, and SRp55, and modulates their function in the regulation of tau exon 10 inclusion *in vitro* ^17–19^. In human DS brain, the protein level of 3R-tau is increased and correlated with the Dyrk1A level^17^. Whether Dyrk1A overexpression causes changes in 3R-tau and 4R-tau resulting from endogenous tau exon 10 splicing *in vivo* is not determined.

In the present study, we determined the alternative splicing of endogenous tau exon 10 in differentiated human neuronal progenitor cells and in Ts65Dn mice, which are a commonly used mouse model of DS and contain one extra copy of Dyrk1A gene, and investigated the role of Dyrk1A in tau exon 10 splicing. We found that inhibition of Dyrk1A by harmine or green tea flavonol epigallocatechin-gallate (EGCG) promotes 4R-tau expression. The alternative splicing of tau exon 10 is dysregulated, leading to a decrease in the 4R-tau/3R-tau ratio in the brains of Ts65Dn mice. Importantly, inhibition of Dyrk1A by EGCG rescued the dysregulated 4R-tau/3R-tau ratio and improved the impaired general behaviors in Ts65Dn mice.

## Results

### 4R-tau has a higher affinity than 3R-tau to bind to microtubules

**Figure 1:**
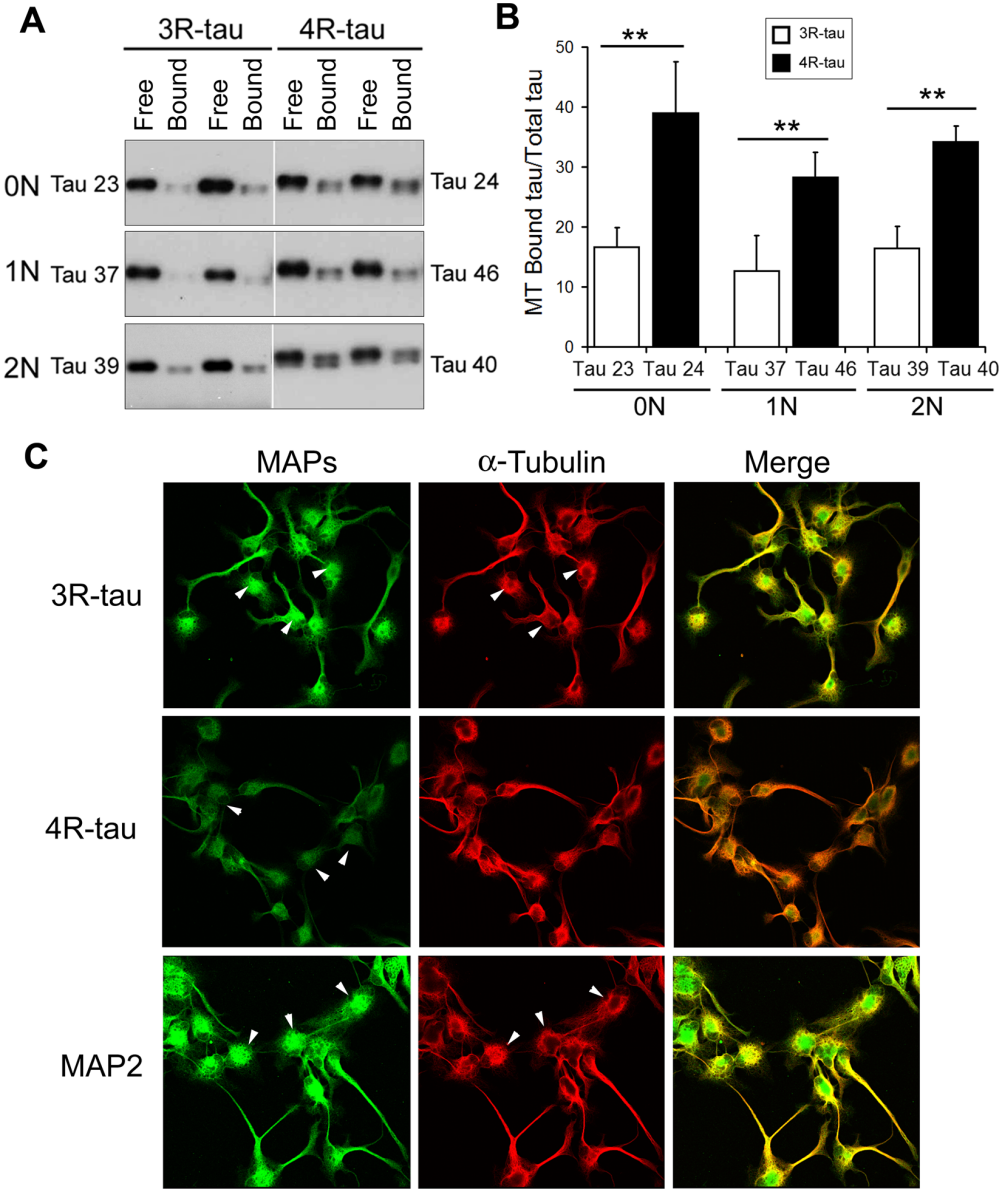
Association of 4R-tau with microtubules is stronger than 3R-tau. (**A,B**) Six isoforms of tau fused with GFP at the N-terminus were overexpressed in N2a cells for 2 days. The cells were incubated with microtubule-stabilizing buffer, and the soluble proteins and insoluble proteins were subjected to Western blots to measure the relative levels of tau isoforms in both soluble (free tau) and insoluble (microtubule bound tau, MT-tau) fractions (**A**). The blots were quantitated by densitometry and the ratios of MT-tau/total-tau are presented as mean ± S.D. (n = 3) (**B**). *p < 0.01. (**C**) Human neuronal progenitor cells were differentiated for 6 days with retinoid acid, and then subjected to immunostaining with anti-3R-tau, anti-4R-tau, anti-MAP2 or anti-α-tubulin followed by fluorescence conjugated anti-bodies.

The major biological function of tau is to stimulate microtubule assembly and stabilize microtubule structure. Adult human brain expresses six isoforms of tau — tau 23 (0N3R), tau 24 (0N4R), tau37 (1N3R), tau46 (1N4R), tau 39 (2N3R) and tau 40 (2N4R) — by the alternative splicing of the exons 2,3, and 10 of its pre-mRNA. To learn the biological consequence of tau exon 10 splicing, we studied the binding ability of 3R-tau and 4R-tau to microtubule. We overexpressed six human tau isoforms targeted with GFP at the N-terminus in N2a cells, separated microtubule (MT)-bound and unbound tau, and analyzed their levels by Western blots. We found that most of tau existed in MT-unbound fraction under this assay condition (Fig. 1A). The ratios of 3R-taus (tau23, tau37, and tau39) in MT-unbound fraction over total (MT-bound and unbound fractions) were less than that of 4R-taus (tau24, tau46, and tau40) (Fig. 1B). These results suggest that 4R-tau has a higher affinity than 3R-tau to bind to microtubule.

To learn subcellular localization of 3R-tau and 4R-tau, we differentiated human neuronal progenitor as described above, then double-immunostained with antibodies against 3R-tau or 4R-tau and tubulin. We observed that both 3R-tau and 4R-tau showed somatic and neuritic expression (Fig. 1C). However, we observed that 3R-tau is mainly localized in the soma of the cells, whereas 4R-tau showed similar levels in the soma and the neurites and co-localized better with tubulin (Fig. 1C). These results further support that 4R-tau associated with microtubule stronger than 3R-tau.

### Dyrk1A regulates the alternative splicing of tau exon 10

**Figure 2:**
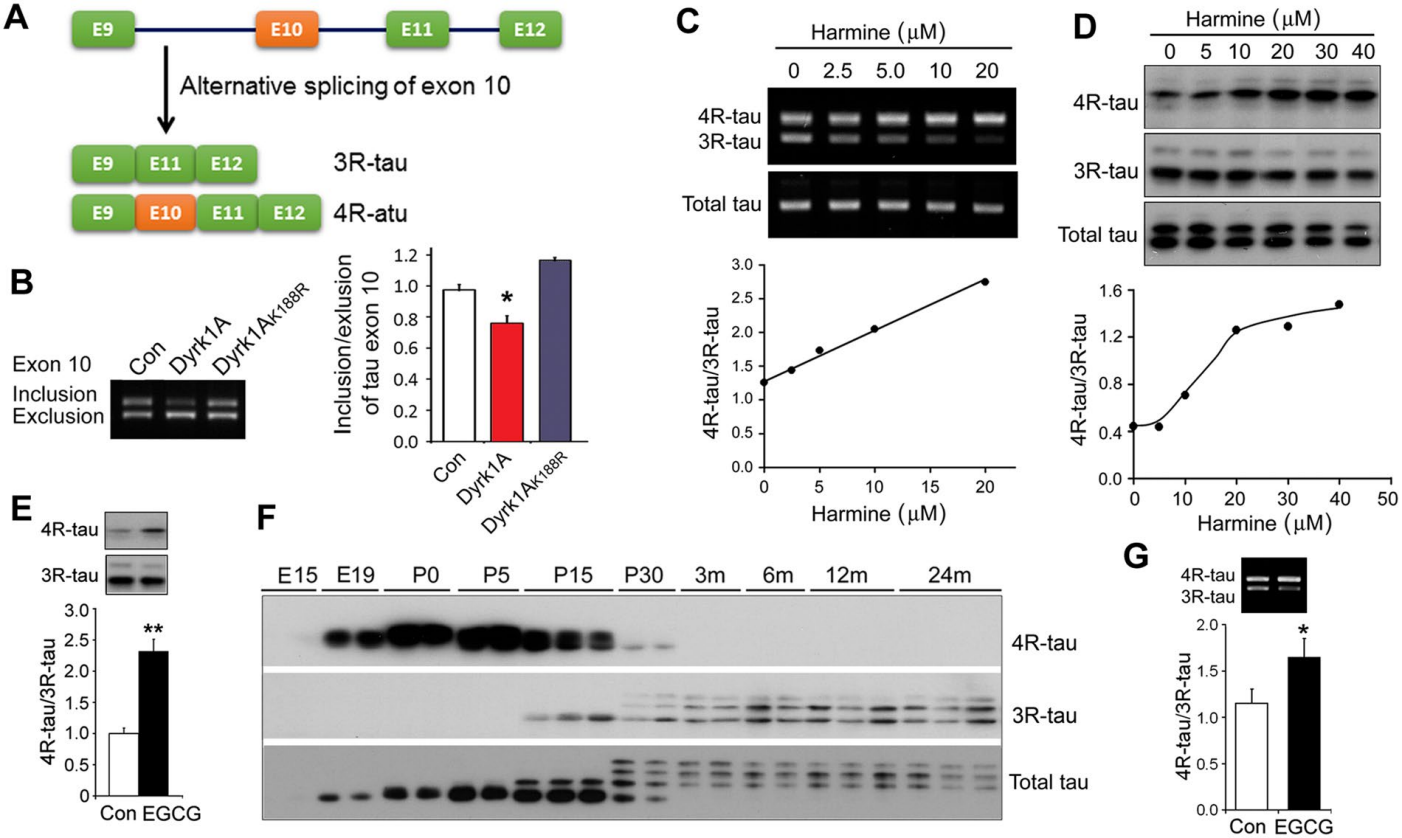
Dyrk1A suppresses tau exon 10 inclusion. (**A**) Diagram of 3R-tau and 4R-tau generated by alternative splicing of tau exon 10. (**B**) Dyrk1A and Dyrk1A_K188R_ were co-transfected into HEK-293FT cells with pCI/SI9-LI10 and splicing products of tau exon 10 were analyzed by RT-PCR. The ratio of inclusion/exclusion of tau exon 10 was calculated. (**C,D**) Differentiated human neuronal progenitor cells by retinoid acid for six days were treated with various concentration of harmine for 24 hrs. The expression of 3R-tau, 4R-tau or total tau mRNA and protein were analyzed by RT-PCR (**C**) and Western blots (**D**) developed with anti-3R-tau (RD3), anti-4R-tau (RD4) and anti-total tau (92e), respectively. The ratio of 4R-tau/3R-tau at mRNA (**C**) or at protein (**D**) levels was plotted against the concentration of harmine. (**E**) Differentiated human neuronal progenitor cells as described above were treated with 20 μM EGCG for 24 hr. 3R-tau and 4R-tau were analyzed by Western blots. (**F**) Forebrains were collected from Wistar rats at embryonic 15 day (E15), E19, postnatal day 0 (P0), P5, P15, P30, postnatal 3 month (3 m), 6 m, 12 m, and 24 m. Expression of 3R-tau, 4R-tau and total tau were analyzed by Western blots. (**G**) Neonatal Sprague-Dawley rats at postnatal day 10 were injected intracerebroventricularly (ICV) with 2 μl of 1 mM EGCG. The mRNAs of 3R-tau and 4R-tau in the forebrains were analyzed by RT-PCR 48 hr post-ICV injection. The ratios of 4R-tau/3R-tau were calculated. The data are presented as mean ± S.D. (n = 3–5); *p < 0.05.

Splicing factors, ASF, SRp55, SC35, and 9G8, regulate the alternative splicing of tau exon 10 and Dyrk1A phosphorylates them and modulates their function in tau exon 10 splicing^17, 18^. To confirmed the role of Dyrk1A in tau exon 10 splicing, we co-expressed Dyrk1A with mini-tau gene pCI/SI9-LI10 that consists of tau exons 9, 10 and 11 and partial intron 9 and full length of intron 10 (Fig. 2A) in HEK-293FT cells and measured the splicing products of tau exon 10 by RT-PCR. Consistent with previous study, we found that overexpression of Dyrk1A significantly promoted tau exon 10 exclusion (Fig. 2B), but overexpression of Dyrk1A_K188R_, a dead enzyme, failed to promote tau exon 10 exclusion (Fig. 2B). Thus, these results conform that Dyrk1A suppresses tau exon 10 inclusion.

To investigate whether alternative splicing of endogenous tau exon 10 is regulated by Dyrk1A, we treated differentiated human neuronal progenitor cells with various concentrations of harmine, a specific Dyrk1A inhibitor^20^ for 24 hr and then analyzed both the mRNA and the protein levels of 3R-tau and 4R-tau by RT-PCR and Western blots, respectively. After differentiated with 10 μM retinoid acid for 6 days, these cells expressed both 3R-tau and 4R-tau (Fig. 2C,D). Inhibition of Dyrk1A with harmine significantly suppressed the expression of 3R-tau and promoted the expression of 4R-tau, resulting in increases of 4R-tau/3R-tau ratio at both mRNA (Fig. 2C) and protein (Fig. 2D) levels in a dose-dependent manner. These data indicate that inhibition of Dyrk1A promotes tau exon 10 inclusion, leading to an increase in 4R-tau expression and a decrease in 3R-tau expression.

Green tea flavonol epigallocatechin-gallate (EGCG) is another potent and selective inhibitor of Dyrk1A^21^. We found that 20 μM EGCG treatment for 24 hr enhanced tau exon 10 inclusion and 4R-tau expression in above differentiated human progenitor cells (Fig. 2E), supporting the role of Dyrk1A in tau exon 10 inclusion.

Alternative splicing of tau exon 10 is regulated during development in both human and rodent brain^9, 22^. Fetal and newborn rodents express 3R-tau only and adult rodents mainly express 4R-tau^9, 22^. To find time window for the study of tau exon 10 splicing, we determined the expression patterns of 3R-tau and 4R-tau in rat forebrain during development by Western blots. We found that rat brains expressed certain amount of 3R-tau and 4R-tau at around postnatal day 15 (Fig. 2F). To learn the effect of EGCG on tau exon 10 splicing *in vivo*, thus, we injected EGCG into the cerebral ventricles of P10 rats and then measured the splicing products of tau exon 10 two days post injection. We found that EGCG treament significantly promoted tau exon 10 inclusion, leading to increased 4R-tau/3R-tau ratio (Fig. 2G). Taken together, these results suggest that Dyrk1A suppresses tau exon 10 inclusion, enhances 3R-tau and suppressed 4R-tau expression.

### Developmental expression of 3R-tau and 4R-tau is dysregulated in the brains of Ts65Dn mice

**Figure 3:**
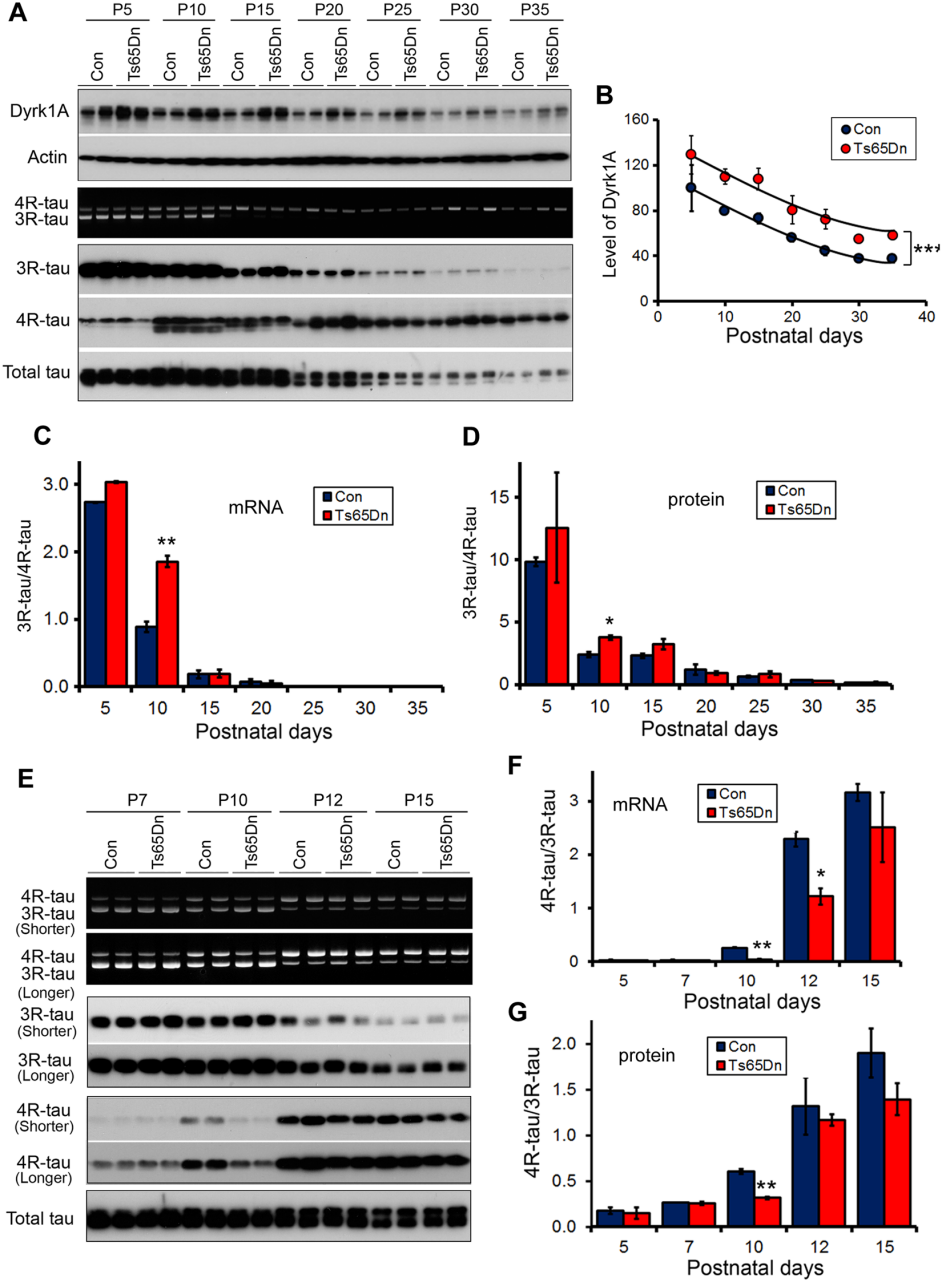
The alternative splicing of Tau exon 10 is dysregulated in neonatal Ts65Dn mouse brains. (**A–D**) Forebrains were collected from Ts65Dn mice and the littermates at P5, P10, P15, P20, P25, P30, and P35. Dyrk1A, 3R-tau, 4R-tau and total tau were analyzed by RT-PCR for mRNA and by Western blots for protein developed with anti-Dyrk1A (8D9), anti-3R-tau (RD3), anti-4R-tau (RD4), and anti-total tau (92e). The levels of Dyrk1A (**B**) and the ratios of 3R-tau/4R-tau at the mRNA (**C**) and the protein (**D**) were calculated. (**E–G**) Mouse forebrains from Ts65Dn and the littermates at P5, P7, P10, P12, and P15 were analyzed 3R-tau, 4R-tau, and total tau by RT-PCR and Western blots with shorter exposure or longer exposure (**E**). The ratios of 3R-tau/4R-tau at the mRNA (**F**) and the protein (**G**) levels were calculated. The Data are presented as mean ± S.D. (n = 3–5) and statistically analyzed with two-way ANOVA followed by Bonferroni post-test. The difference between Ts65Dn and the littermate controls at postnatal day 10 (P10) was analyzed by paired student *t* test. *p < 0.05, **p < 0.01, and ***p < 0.001.

Individuals with Down syndrome develop early onset tau pathology^11^. Dyrk1A is overexpressed in DS brain. To determine the role of Dyrk1A overexpression in tau exon 10 splicing *in vivo*, we analyzed tau exon 10 splicing in Ts65Dn mice during development since the adult rodents mainly express 4R-tau. We confirmed that the extra gene dosage resulted in the overexpression of Dyrk1A in Ts65Dn brains at all ages studied from postnatal day 5 (P5) to P35, although the expression of Dyrk1A was found to decrease gradually from P5 to P35 in both Ts65Dn and control mice (Fig. 3A,B). The tau isoforms in mouse forebrain were shifted from 3R-tau to 4R-tau during P5 to P30 (Fig. 3A). Comparing the alternative splicing of tau exon 10 between Ts65Dn mice and control littermates, we found an increase of 3R-tau and a decrease of 4R-tau at both mRNA and protein levels at P10 in Ts65Dn mouse brains (Fig. 3A). To clearly show the changes in the products of tau exon 10, we calculated the ratio of 3R-tau and 4R-tau. We found that the ratio of 3R-tau/4R-tau was significantly increased in the Ts65Dn mouse brains as compared with control mouse brain at P10 at the mRNA (Fig. 3C) as well as at the protein (Fig. 3D) levels. Thus, the shifting from 3R-tau to 4R-tau is significantly delayed in Ts65Dn brain (Fig. 3A,C,D), and the developmental regulation of tau exon 10 splicing is dysregulated in the brains of Ts65Dn mice.

To learn the detail of the dysregulation of tau exon 10 splicing in Ts65Dn, we added other time points, P7 and P12, and measured the expression of 3R-tau and 4R-tau in another cohort of animals. We found that compared with the control littermates, the expression of 3R-tau was increased and 4R-tau was decreased in Ts65Dn mice at both mRNA and/or protein (Fig. 3E) levels at P7, P10, P12 and P15, but the changes were peaked at P10 in which was showed by the ratio of 4R-tau/3R-tau better at mRNA (Fig. 3F) and protein (Fig. 3G). These results further support that tau exon 10 is dysregulated in Ts65Dn mice and overexpression of Dyrk1A enhanced 3R-tau and suppressed 4R-tau expression.

### Ts65Dn mice display abnormal general behavior and learning and memory

**Figure 4:**
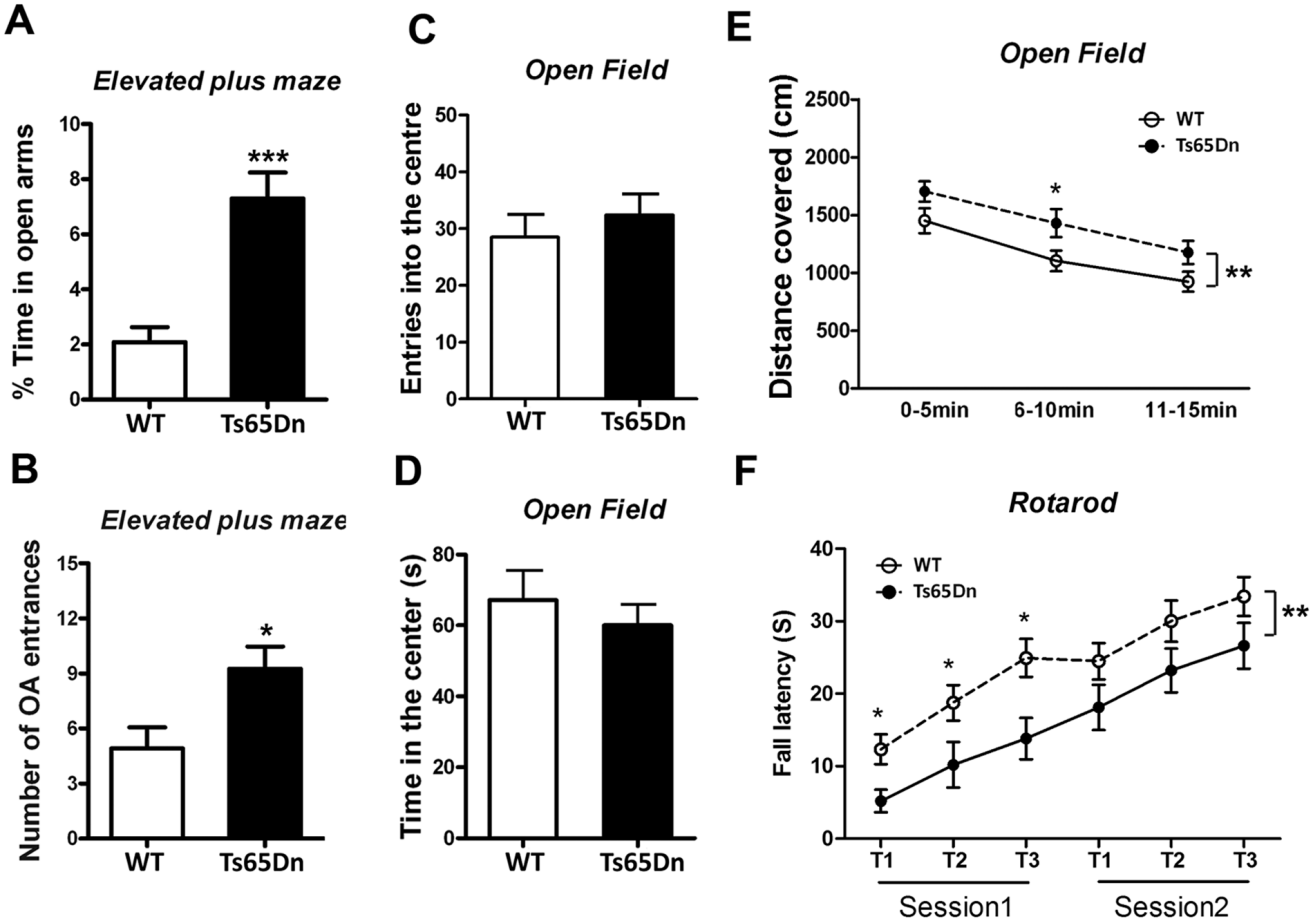
Ts65Dn mice show abnormal general behavior. (**A,B**) Male Ts65Dn and the control mice were tested with elevated plus maze. The amount of time (**A**) spent in the open arm and number (**B**) of open arm (OA) entrances were analyzed. (**C–E**) Male Ts65Dn and the control mice were subjected for open field test. Enters into the center area (**C**), time amount spent in center area (**D**), and total distance covered (**E**) were analyzed. (**F**) Male Ts65Dn and control mice were analyzed locomotor activity by rotarod test. The data are presented as mean ± SEM and statistically analyzed with student t test (**A–D**) and with two way ANOVA followed by Bonferroni post-test (**E,F**) (n = 11–12). *p < 0.05; ***p < 0.001.

To investigate whether Ts65Dn mice, in which developmental tau exon 10 splicing is dysregulated, display abnormal general behavior, we first evaluated the anxiety with the elevated plus-maze task and open-field task in Ts65Dn and controls at 2.5 month old. In the evaluated plus-maze test, Ts65Dn mice spent longer time in the open arms (Fig. 4A) and entered the open arms more times than the control mice (Fig. 4B), suggesting less anxiety in Ts65Dn mice. In the open field test, Ts65Dn mice spent similar time in the center zone and showed similar entries into the center as the controls (Fig. 4C,D). However, the total distance traveled by Ts65Dn mice in the area was longer than the control mice during all sessions, as analyzed in three intervals of 5 min each (Fig. 4E). We used accelerating rotarod test to determine the locomotivity and motor coordination. We observed that Ts65Dn mice displayed less latency to fall compared with the controls (Fig. 4F), suggesting the deficit in locomotivity and motor coordination in Ts65Dn mice. Thus, Ts65Dn mice show less anxiety, increased activity and deficit in locomotivity and motor coordination.

**Figure 5:**
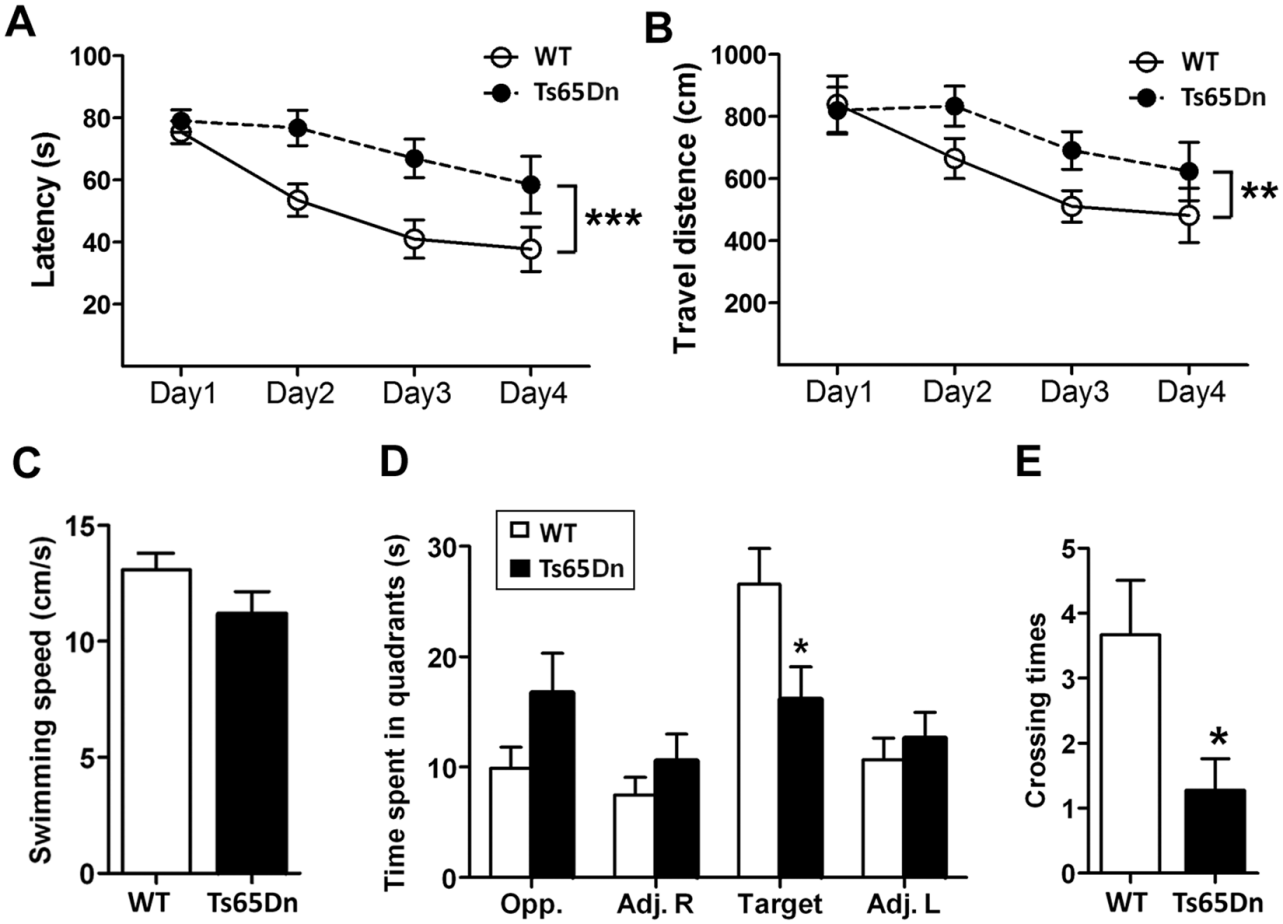
Learning and memory are impaired in Ts65Dn mice. Male Ts65Dn and control mice were subjected to spatial reference memory by Morris water maze. During the training phase, traveled time amount (**A**), distance (**B**) to reach the platform, and swimming velocity (**C**) were analyzed. In the probe trial, the time amount spent in the four quadrants (**D**) and crossing numbers in platform zone were analyzed. The data are presented as mean ± SEM (n = 11–12); *p < 0.05; **p < 0.01.

Then, we studied the learning and memory of Ts65Dn mice by using Morris Water Maze test at age of 5 months. Performance of animals during training was analyzed as latency and traveled distance to reach the submerged platform. Both Ts65Dn and control mice spend less time and travel shorter distance to reach the platform at day 4 than at day 1 (Fig. 5A,B), suggesting they are able to learn. Compared with the control mice, Ts65Dn spent more time (Fig. 5A) and traveled longer distance (Fig. 5B) than controls to reach the platform during training phase, suggesting an impairment of spatial learning in Ts65Dn. The swimming speed of these two groups of mice was not different, eliminating the bias of travel times and distances in case of different swim speeds (Fig. 5C).

To assess the spatial memory of the mice, we carried out probe trials one day after the training trials to assess the spatial memory of the mice. On the probe trials, Ts65Dn spent less time in the target quadrant (Fig. 5D) and showed less than wild type mice on the number of crossings of the platform zone (Fig. 5E). Thus, the spatial memory is also impaired in Ts65Dn mice.

### EGCG attenuates the increased 3R-tau and impaired behaviors in Ts65Dn mice

**Figure 6:**
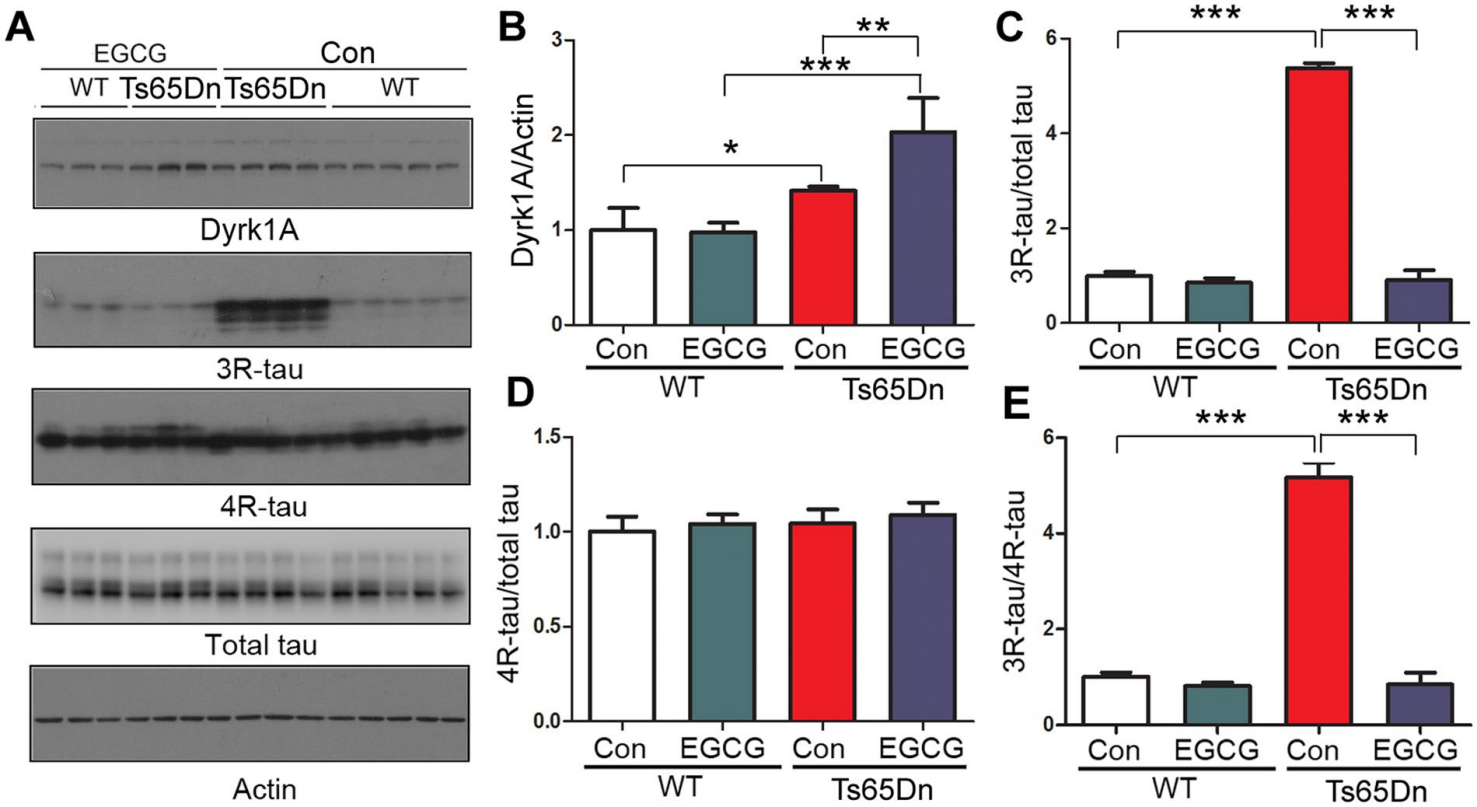
EGCG suppresses the increased 3R-tau expression in Ts65Dn mice. Ts65Dn or control mice were treated with EGCG from mating to adulthood. The 3R-tau, 4R-tau and total tau in the forebrains from 5 month old mice were analyzed by Western blots (**A**). The level of Dyrk1A (**B**), 3R-tau (**C**), 4R-tau (**D**) and ratio of 3R-tau/4R-tau (**E**) are presented as mean ± S.D. (n = 3–5) and analyzed by two way ANOVA followed post-hot test. *p < 0.05; **p < 0.01; ***p < 0.001.

EGCG can cross the blood-brain barrier^23, 24^ and the placental barrier^25^. To learn whether the inhibition of Dyrk1A by EGCG could attenuate the dysregulated tau exon 10 splicing in Ts65Dn, we adminstrated EGCG to mice in drinking water from gestation (started during initial mating period) to 5 months old, and then analyzed the levels of Dyrk1A, 3R-tau, 4R-tau and total tau by Western blots. We found that the level of Dyrk1A in EGCG treated Ts65Dn mouse brains was further increased (Fig. 6A,B). Level of 3R-tau (Fig. 6A,C), but not 4R-tau (Fig. 6A,D), was increased in Ts65Dn, leading to increased ratio of 3R-tau/4R-tau (Fig. 6E). Interestingly, the increased 3R-tau level and the ratio of 3R-tau/4R-tau in Ts65Dn mice were attenuated by EGCG treatment (Fig. 6A,C–E). These results suggest that dysregulated tau exon 10 splicing in Ts65Dn mice may be the result from Dyrk1A overexpression.

**Figure 7:**
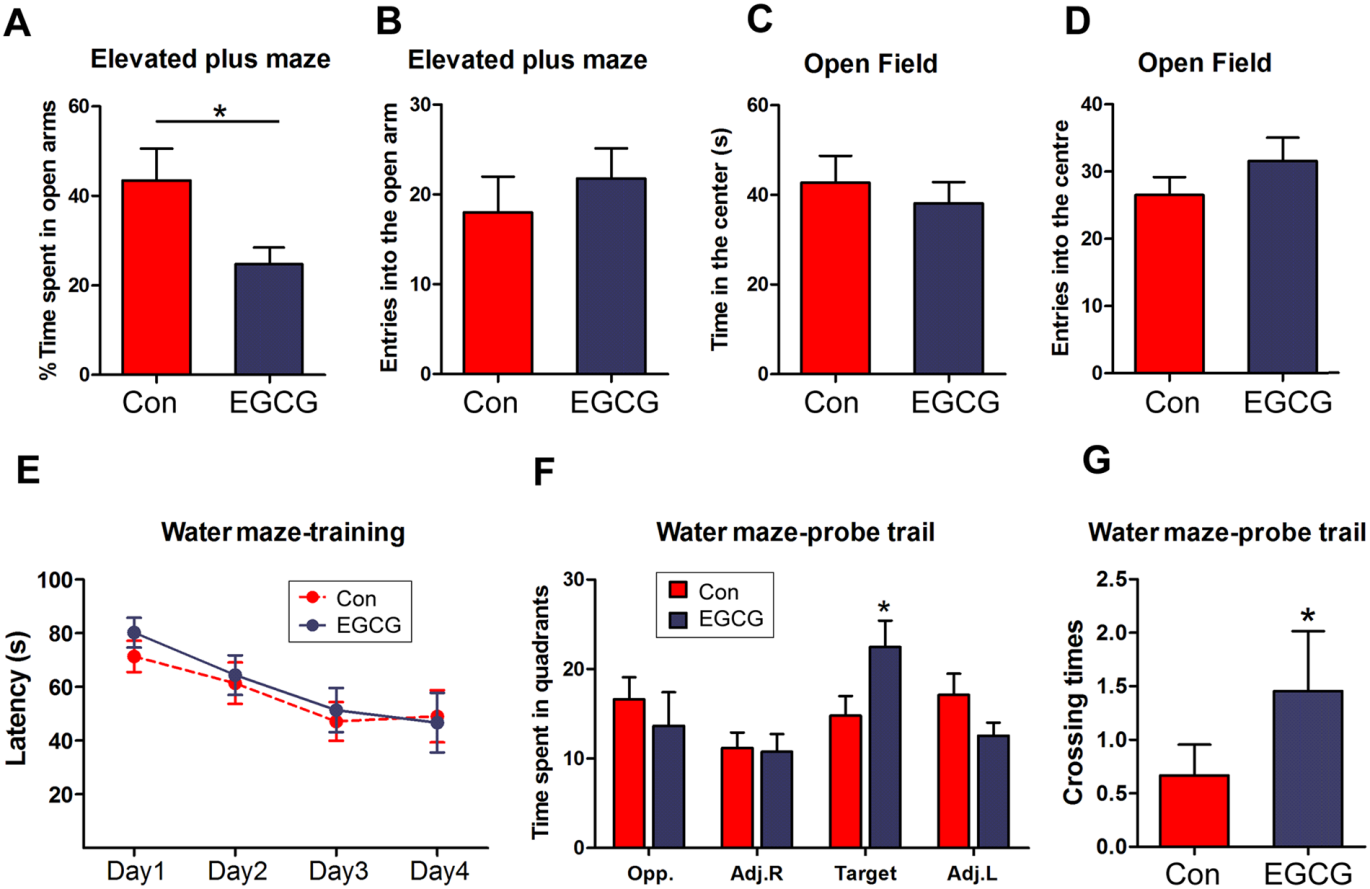
EGCG rescues the impaired behavior of Ts65Dn. Ts65Dn mice were treated with EGCG treatment from prenatal to adulthood and subjected to elevated plus maze (**A,B**), open field test (**C,D**), and Morris water maze test (**E–G**). The data were presented as mean ± SEM (n = 9–13) and analyzed by student *t* test. *p < 0.05.

To investigate the role of Dyrk1A inhibition in above behavioral deficits in Ts65Dn mice, we tested the mouse behaviors in above EGCG admistrated mice. We found that EGCG treated Ts65Dn stayed less time in open arms than control treated Ts65Dn (Fig. 7A), suggesting EGCG rescued the decreased anxiety. EGCG did not alter the learning ability of Ts65Dn, but EGCG treated mice spent more time in target quadrant (Fig. 7F) and crossed the target quadrant more times than control littermates (Fig. 7G), suggesting EGCG attenuates the memory deficit in Ts65Dn. These results suggest that Dyrk1A overexpression in Ts65Dn mice may contribute to the behavioral deficits of the mice.

## Discussion

Adult human brain expresses approximately equal levels of 3R-tau and 4R-tau. Mutations of tau, in which this balance is disrupted, cause neurodegenerative disorders. In addition to the inherited frontal lobe dementia, imbalanced expression of 3R-tau and 4R-tau has been seen in sporadic tauopathies. Thus, equal levels of 3R-tau and 4R-tau are required for maintaining normal brain function. Dysregulation of tau exon 10 is sufficient to cause some of tauopathies. In the present study, we found that inhibition of Dyrk1A by harmine or EGCG enhanced tau exon 10 inclusion and 4R-tau expression in differentiated neuronal progenitor cells and in neonatal rat brains, leading to increase of ratio of 4R-tau and 3R-tau. Alternative splicing of tau exon 10 is dysregulated in neonatal Ts65Dn mouse brain. In these animals, 3R-tau was increased and 4R-tau was decreased. Ts65Dn mice show less anxiety, poor locomotivity and motor coordination, and learning and memory deficits. Treatment with EGCG from green tea extract not only ameliorates the increased 3R-tau expression, but also attenuates the decreased anxiety and impaired memory in Ts65Dn mouse brains, indicating green tea may be beneficial for the DS and 3R-tau related tauopathies.

Individuals with DS develop AD pathologies, Amyloid β plaques and neurofibrillary tangles during the 4^th^ decade of life. Aβ pathology may be caused by overexpression of amyloid β precursor protein (APP), by which tau becomes pathogenic remains unclear. We previously found that Dyrk1A on one hand phosphorylates tau and makes tau become better substrate for GSK-3β^26^ and on other hand it phosphorylates splicing factors, such as ASF, SC35 and SRp55, and suppresses their function^17–19^. Overexpression of Dyrk1A is correlated positively with 3R-tau level and negatively with 4R-tau level in DS brain^17^. Here, we found for first time that tau exon 10 splicing is dysregulated in neonatal Ts65Dn mouse brains. Thus, overexpression of Dyrk1A in DS may contribute to tau pathology in DS by phosphorylation of tau and dysregulation of tau exon 10 splicing.

3R-tau and 4R-tau are resulted from the alternative splicing of tau exon 10, in which SR proteins are involved^27^. ASF and SC35 are important splicing factors to promote tau exon 10 inclusion^17, 18, 28^. They act on the polypurine enhancer and SC35-like enhancer of 5′ exon 10^17, 18, 29^. The function of ASF and SC35 are regulated by phosphorylation. Cdc2-like kinase and SR protein kinase phosphorylate ASF and drive it from the cytoplasm into the nucleus and from speckles into nascent transcripts, respectively^30, 31^ and enhances its function. Whereas Dyrk1A phosphorylates ASF at Ser-227, -234, and -238, and leads it into speckles and suppresses its function to promote tau exon 10 inclusion^17^. Since adult rodent brains mainly express 4R-tau, we employed neonatal mouse and rat brains to study the role of Dyrk1A. We found that 3R-tau is increased and 4R-tau is decreased, resulting in decrease of ratio of 4R-tau and 3R-tau at both mRNA and protein levels in neonatal Ts65Dn mice at postnatal day 7 to day 12, in which relative level of 3R-tau and 4R-tau are expressed. Inhibition of Dyrk1A by either harmine or EGCG suppresses endogenous tau exon 10 inclusion in cultured cells or in rat and Ts65Dn mouse brains. Thus, we here provide a direct evidence that Dyrk1A participates the regulation of tau exon 10 splicing and increases 3R-tau/4R-tau ratio *in vivo*.

Different from DS in which Dyrk1A is overexpressed as a results of an extra copy of the gene, Dyrk1A is truncated at C-terminus and correlated with the activation of calpain I in AD brain^32^. Calpain I proteolyzes Dyrk1A at the C-terminus and enhances its kinase activity toward to tau *in vitro* ^32^. In AD brain, truncation of Dyrk1A is positively correlated with the 3R-tau/4R-tau ratio. Thus, negative correlation between truncation of Dyrk1A and ratio of 4R-tau and 3R-tau in AD brain may also be contributed by up-regulation of Dyrk1A.

Dyrk1A is located at the Down syndrome critical region. Dyrk1A transgenic mice show phenotype of Down syndrome, including learning and memory deficits^33–35^. Dyrk1A is predominantly expressed in the speckles of nucleus^14^, a structure which represents the splicing factor compartment^14, 36^, and regulates splicing of pre-mRNA^15, 16^. Engineering Dyrk1A over dosage yields Down syndrome-characteristic cortical splicing aberrations^37^. Knockdown of Dyrk1A expression by AAV2/1-shDyrk1A attenuates hippocampal-dependent defects in the Ts65Dn mice^38^. Thus, overexpression of Dyrk1A is pivotal in the learning and memory deficits of Ts65Dn. EGCG, a key bioactive ingredient of green tea, is a Dyrk1A inhibitor. In the present study, we found that the Ts65Dn mice display abnormal general behavior and impaired learning and memory. In addition to rescue the dysregulated tau exon 10 splicing, EGCG rescues the anxiety and improves the memory of Ts65Dn. Thus, overexpression of Dyrk1A may contribute to the reduced anxiety and learning and memory deficit in Ts65Dn mice. Since Dyrk1A phosphorylates numerous proteins, whether these behavior abnormalities in Ts65Dn mice are mediated by dysregulation of tau exon 10 remains elusive. Previous studies also reported the rescue of cognitive deficits in Down syndrome mouse models and in humans by EGCG treatment^39^. Thus, EGCG may have beneficial effects for DS and AD.

In summary, this study provides a direct evidence that Dyrk1A involved in the regulation of alternative splicing of tau exon 10 *in vivo*. Overexpression of Dyrk1A in neonatal Ts65Dn mouse brains suppressed tau exon 10 inclusion, leading to an increase of 3R-tau/4R-tau ratio. Inhibition of Dyrk1A promotes endogenous tau exon 10 inclusion in cultured cells and in neonatal rat brain. Treatment with Dyrk1A inhibitor EGCG suppressed 3R-tau expression and attenuated anxiety and memory deficits in Ts65Dn mice. Thus, up-regulation of Dyrk1A may contribute to tau pathogenesis via dysregulation of tau exon 10 splicing in DS and AD. Dyrk1A might be an ideal therapeutic target for DS and AD. EGCG may have potential effect on the treatment or prevention of these diseases.

## Materials and Methods

### Animals

Sprague-Dawley rats were from Model Animal Research Center of Nanjing University (Nanjing, Jiangsu, China). Wistar rats were from Charles River Laboratories, Inc. (Wilmington, MA, USA). Ts65Dn mice and control mice were obtained from Jackson Laboratory (Bar Harbor, ME). The animals were housed in a 12-hour light/dark schedule with free access to food and water. Pregnant Wistar female rats were killed at 15 and 19 days of gestation, and the brains of rat fetuses (E15 and E19) were dissected immediately. Wistar rat brains were collected from pups on the day of birth (P0), and male rats at post-natal day 5 (P5d), P15d, post-natal month 1 (P1m), P6m, P12m, and P24m. Ts65Dn or the littermate brains were also collected from mouse at postnatal day 5 (P5), P7, P10, P12, P15, P20, P25, P30, and P35. The housing, breeding, and animal experiments were in accordance with the approved protocols from the Institutional Animal Care and Use Committees of New York State Institute for Basic Research in Developmental Disabilities (revised on October 22, 2010) and Nantong University (approved on July 20, 2012) according to the PHS Policy on Human Care and Use of Laboratory animals.

### Plasmids, Proteins, and Antibodies

Recombinant rat Dyrk1A and mammalian expression vector pcDNA3 containing either rat Dyrk1A or kinase-dead Dyrk1A (Dyrk1A_K188R_) were gifts from Dr. Yu-Wen Hwang from our institute. pEGFP/tau23, pEGFP/tau24, pEGFP/tau37, pEGFP/tau39, pEGFP/tau40, and pEGFP/tau46 were reconstructed from the prokaryotic plasmids received as gifts from Dr. Michel Goedert (Molecular Biology Unit, Medical Research Council, Cambridge, UK). pCEP4-SC35-HA and pCEP4-ASF-HA were gifts from Dr. Tarn of the Institute of Biomedical Sciences, Academia Sinica, Taiwan. pCI-SI9/LI10 containing a tau mini-gene, SI9/LI10, comprising tau exons 9, 10, and 11, part of intron 9, and the full length of intron 10 was kindly provided by Dr. Jianhua Zhou from University of Massachusetts Medical School, Worcester, Massachusetts. Monoclonal antibody 8D9 was raised against a histidine-tagged protein containing the first 160 residues of rat Dyrk1A^40^. The monoclonal anti-β-tubulin and anti-α-actin were bought from Sigma (St. Louis, MO). Monoclonal anti-3R-tau (RD3) and anti-4R-tau (RD4) were from Upstate Biotechnology (Lake Placid, NY). Monoclonal anti-ASF was from Santa Cruz (Santa Cruz, CA). Polyclonal anti-tau R134d and 92e were described previously^41^. Peroxidase-conjugated anti-mouse and anti-rabbit IgG were obtained from Jackson ImmunoResearch Laboratories (West Grove, PA); Alexa Fluor 555-conjugated goat anti-rabbit IgG, and Oregon Green 488-conjugated goat anti-mouse IgG were from Life Technologies. The ECL kit was from Thermo Fisher Scientific (Waltham, MA).

### Cell Culture and Transfection

HEK-293FT cells were maintained in Dulbecco’s modified Eagle’s medium supplemented with 10% fetal bovine serum (Invitrogen, Carsbad, CA) at 37 °C. Normal human neuronal progenitor cells (Lonza, Walkersville, MD) were maintained in Neurobasal supplemented with 2% B27 (Invitrogen), 20 ng/ml fibroblast growth factor 2 (FGF-2), 20 ng/ml epidermal growth factor, and 10 ng/ml leukemia inhibitory factor and differentiated with 10 μM retinoid acid in the maintenance medium for 6 days. Transfections were performed with Lipofectamine 2000 (Invitrogen) or FuGene 6 (Roche, Indianapolis, IN), according to the manufactures’ instructions.

### Western blot analyses

Cultured cells were lysate with Laemmli buffer. Mouse forebrain tissue was homogenized in 9 volumes of buffer containing 50 mM Tris-HCl, pH 7.4, 8.5% sucrose, 10 mM β-mercaptoethanol, and 2.0 mM EDTA and added 2-fold concentrated Laemmli buffer. The cells lysates or the brain homogenates were boiled for 5 min and the protein concentration was measured by using modified Lowry. The same amounts of protein from each sample were separated by sodium dodecyl sulfate (SDS)–polyacrylamide gel electrophoresis (PAGE) and electro-blotted onto PVDF membrane. After blocking with 5% fat-free milk, the membrane was incubated with primary antibodies, such as anti-3R-tau (1:2000), anti-4R-tau (1:500), anti-Dyrk1A (1:10000), anti-GAPDH (1:2000), or anti-actin (1:4000) overnight at room temperature in the presence of 0.1% NaN_3_. After washing with TBST (Tris-HCl, pH 7.4, 150 mM NaCl, 0.05% Tween 20) three times, the membrane was incubated with the corresponding HRP-conjugated secondary antibody for ~2 h. After washing with TBST, the blot was visualized by enhanced chemiluminescence (Thermo Scientific, Rockford, IL) and quantified by densitometry using the Multi Gauge V3.0 software (Fuji Photo Film Co., Ltd).

### Quantitation of tau exon 10 splicing by reverse transcription-PCR (RT-PCR)

Total RNA was isolated from cultured cells or from mouse brains by using an RNeasy mini kit (Qiagen GmbHHilden, Germany). Six hundred ng of total RNA was used for first-strand cDNA synthesis with oligo (dT)18 by using an Omniscript reverse transcription kit (Qiagen GmbH). PCR was performed by using Prime- STAR^TM^ HS DNA Polymerase (Takara Bio Inc., Otsu, Shiga, Japan) with primers (forward 5′GGTGTCCACTCCCAGTTCAA3′ and reverse 5′CCCTGGTTTATGATGGATGTTGCCTAATGAG3′) for transfected pCI/SI9-SI10, and with primers (forward 5′AACACCGCCCACCCGGGAG3′ and reverse 5′GTCTGTCTTGGCTTTGGCATTCTC3′) for endogenous mouse tau to measure alternative splicing of tau exon 10 under conditions: denaturation for 5 min at 98 °C was followed by 30 cycles with denaturation for 10 sec at 98 °C, annealing for 15 sec at 55 °C, polymerization for 30 sec at 72 °C and a final extension for 10 min at 72 °C. The PCR products were resolved on 1.5% agarose gels and quantitated using the Molecular Imager system (Bio-Rad, Hercules, CA).

### Fractionation of microtubule association tau

HEK-293FT cells were transfected with six isoforms of tau respectively. The cells were harvested with microtubule stabilization buffer^42^ and incubated for 8 min at 32 °C. The microtubule association fraction was sedimented by 15,000 × g centrifugation for 10 min at room temperature. Microtubule-binding tau and free tau were analyzed by Western blots.

### Intracerebroventricular Injection

Sprague-Dawley rats at postnatal day 10 (P10) were first anesthetized by wrapping in ice pack for 5 min, and then 2 μl of 1 mM EGCG in artificial CSF was injected into the left lateral ventricle of the brain at around 2.5 mm depth. The control animals were treated identically but with vehicle only. The 3R-tau and 4R-tau mRNAs in forebrains were analyzed 2 days after injection by RT-PCR.

### Elevated plus-maze

Ts65Dn and control mice were first subjected for general behavioral test at age of 2.5 months. The elevated plus-maze consisted of four arms (30 × 5 cm) which are connected by a common 5 × 5 cm center area. Two opposite facing arms were open (open arms, OA), whereas the other two facing arms were enclosed by 20 cm height walls (closed arms, CA). The entire plus-maze was elevated on a pedestal to a height of about 80 cm above floor level. During a single 8-min session, an animal was placed onto the central area. Any-maze (Stoelting Co., IL) video tracking system detected the presence of the animal and the time it spent in the different zones. For each animal, the number of CA entries, OA entries, and amount of time spent in CA and OA were recorded. The percentage of time spent in OA and the entries into OA were recorded and calculated to evaluate anxiety-like behavior of animals.

### Open-field activity test

After elevated plus-maze test, the mice were tested by open-field activity. The testing apparatus was a classic open field with a square 50 × 50 cm arena with 40 cm high walls. The mice were individually subjected to the test for 15 min. The arena was divided into 9 equal virtual squares and the general exploration and locomotor activity were recorded. Amount of time spent in the center of the arena and the entries of center arena were recorded as an additional measure of anxiety.

### Accelerating Rota-rod test

The mice were conducted for locomotivity and motor coordination test with accelerating Rota-rod-test by giving each mouse 2 sessions of 3 trials each with the motor in accelerating mode (Ugo BasileSrl, Italy). The rotating speed increased steadily, at a rate of 0.02 cm/s, from 4 to 40 rpm. The latency to fall off the Rota-rod was calculated. Inter-trial intervals were 10–15 min for each mouse.

### Spatial learning and memory task in the water-maze

After all general behavioral tests, the mice were tested spatial learning and memory by Morris water maze at age of 5 months. Morris water maze (MWM) was used to evaluate spatial learning and memory of the mice. The test was performed in a circular white pool (with a diameter of 180 cm and a height of 60 cm) filled with water (23 °C ± 2) made opaque by adding white non-toxic paint. The maze was designated of two principal axes with each line bisecting the maze perpendicular to the other one to divide the maze into four equal quadrants. A platform (13 cm diameter) submersed 1 cm under the water surface was placed in the center of one of the four imaginary quadrants of the tank and maintained in the same position during all trials. Each mouse was given 90 s to find the platform. If the mouse did not find the platform in 90 s, it was gently guided to it. At the end of each trial, the mouse was left on the platform for 20 s. Three such acquisition trials were given on each day for 4 consecutive days. Each mouse performed a total of 12 trials corresponding to a partial training of the spatial reference memory task. A test for retention (probe trial) was given 24 hr after the last day of training. For probe trial, the mouse was allowed to swim in the tank without the escape platform. The swim path, swim distance (cm), escape latency (sec), swim speed (cm/sec), time spent in each quadrant (sec), distance traveled in each quadrant (cm), latency to enter the platform site zone (sec), and the number of platform site zone crossings were recorded through an automated tracking system (Smart video tracking system, Panlab; Harvard Apparatus).

### Treatment of animals with EGCG

Ts65Dn females were administered EGCG in drinking water since from mating to weaning of the pups and then, the pups were fed with EGCG-water till biochemical analyses. EGCG solution was prepared from Mega Green Tea Extract product (Life Extension, USA). As previous study^39^, the EGCG stock solution was prepared as 90 mg/ml and each mouse were put on with drinking water for a dose of ~3 mg per day. Drinking water was refreshed every 3 days. Ts65Dn mice and their controls (2.5 months old) were first submitted to a general behavioral battery, and then cognitive tests were carried out. The mice were sacrificed after cognitive test by cervical dislocation and their forebrains were used for biochemical analyses.

### Statistical analysis

Data were presented as mean ± S.D. or SEM and analyzed by the unpaired two-tailed Student’s *t* test for two groups’ comparison and by one or two way ANOVA for multiple-groups analysis followed by post hoc test or by Bonferroni post-test. *p* < 0.05 was considered statistically significant.
